# Increased Risk of Breast Cancer Associated with CC Genotype of *Has-miR-146a* Rs2910164 Polymorphism in Europeans

**DOI:** 10.1371/journal.pone.0031615

**Published:** 2012-02-20

**Authors:** Hai Lian, Lei Wang, Jingmin Zhang

**Affiliations:** 1 Department of Pathology, The Eleventh Institute of Academy of Military Medical Sciences, Changchun, China; 2 Electrical Diagnosis Division, Chaoyang District People's Hospital, Changchun, China; 3 School of Pharmacy, Jilin University, Changchun, China; Karolinska Institutet, Sweden

## Abstract

**Background:**

Emerging evidence suggests that microRNAs play a critical role in the pathogenesis of breast cancer. Several molecular epidemiological studies were conducted in recent years to evaluate the association between *has-miR-146a* rs2910164 polymorphism and breast cancer risk in diverse populations. However, the results remain conflicting rather than conclusive.

**Methodology/Principal findings:**

We performed a meta-analysis of 6 case-control studies that included 4238 breast-cancer cases and 4469 case-free controls. We assessed the strength of the association, using odds ratios (ORs) with 95% confidence intervals (CIs). Overall, this meta-analysis showed that the rs2910164 polymorphism was not associated with a significantly increased risk of breast cancer in all genetic models (for GC *vs* GG: OR = 1.00, 95% CI = 0.90−1.09, *P*_heterpgeneity_ = 0.364; for CC *vs* GG: OR = 1.16, 95% CI = 0.98−1.36, *P*_heterpgeneity_ = 0.757; for GC+CC *vs* GG: OR = 1.02, 95% CI = 0.93−1.12, *P*_heterpgeneity_ = 0.562; for CC *vs* GC+GG: OR = 1.10, 95% CI = 0.96−1.26, *P*_heterpgeneity_ = 0.441). However, in the stratified analysis by ethnicity, we found the rs2910164 polymorphism was associated with increased breast cancer risk among Europeans in homozygote comparison (CC vs. GG: OR = 1.29, 95%CI = 1.02−1.63, *P*_heterpgeneity_ = 0.950, *P* = 0.032) and recessive model (CC vs. GC+GG: OR = 1.31, 95%CI = 1.05−1.65, *P*_heterpgeneity_ = 0.839, *P* = 0.019). No publication bias was found in the present study.

**Conclusions/Significance:**

This meta-analysis suggests, for the first time, that the CC homozygote of rs2910164 may contribute to breast cancer susceptibility in Europeans.

## Introduction

Breast cancer (BC) is the most frequently diagnosed cancer and the leading cause of cancer death among women globally. About 1.3 million women are diagnosed with BC annually worldwide and about 465,000 die from the disease [1], [2], [3]. In the United States BC is the second most common cause of cancer death in women [1], [3]. Moreover, BC incidence rates have been reported to increase by up to 5% per year in developing countries [2], [3], [4]. Although environmental factors and lifestyle could contribute to the increased BC risk, genetic factors are also implicated in the pathogenesis of the disease. A current priority in BC research is to identify genetic alterations that are directly involved in breast carcinogenesis. To date, a great number of genetic variants have been identified to be potentially associated with BC risk [5], [6], [7], [8]. However, the molecular mechanisms that contribute to breast carcinogenesis remain poorly understood.

Emerging evidence supports a role for microRNAs (miRNAs) in BC development and progression [9], [10]. miRNAs are non-coding, single-stranded RNAs of ∼22 nucleotides and constitute a novel class of gene regulators that are found in both plants and animals. miRNAs are predicted to target over 50% of all human protein-coding genes, thus playing regulatory roles in a variety of physiological and developmental processes [11], [12]. Recent evidence has shown that miRNA mutations or mis-expression are correlated with various human cancers including BC [13], [14]. For example, *microRNA-146a* has been shown to bind to the 3′UTR of the BC susceptibility gene BRCA1 and negatively regulate its expression [15], [16]. A G>C polymorphism (rs2910164), which is located in the sequence of *miR-146a* precursor, results in a change from G:U to C:U in its stem region [16]. Up to now, a few molecular epidemiological studies have investigated the association between the *miR-146a* rs2910164 polymorphism and BC risk [16], [17], [18], [19], [20], [21]. However, the results remain controversial and ambiguous. Because a single study might have been underpowered to detect the overall effects, a quantitative synthesis of the accumulated data from different studies is important to provide evidence on the association of rs2910164 polymorphism with BC risk. Thus, in this study we conducted a meta-analysis to combine all studies available and validate whether the *miR-146a* G>C polymorphisms contribute to BC susceptibility.

## Materials and Methods

### Publication search

We searched the PubMed and Embase databases for all articles on the association between *has-miR146a* rs2910164 polymorphism and BC risk up to September 2011. The following key words were used: “*miR-146a*/rs2910164,” “breast cancer/carcinoma,” and “polymorphism/variant”. The electronic searching was supplemented by checking reference lists from the identified articles and reviews for additional original reports. All the studies must meet the following criteria: (1) case-control study; (2) the outcome had to be breast cancer; and (3) at least two comparison groups (cancer group vs. control group). The major exclusion criteria were: (1) duplicate data, (2) abstract, comment, review and editorial, and (3) no sufficient data were reported.

### Data extraction

Two of the authors (H.L. and L.W.) extracted all data independently, complied with the selection criteria, and reached a consensus on all items. In case of disagreement, a third author (JM.Z.) assessed the articles. The following items were collected: first author's name, year of publication, country of origin, ethnicity, definition of study patients (cases), genotyping method, total number of cases and controls, and genotype distributions in cases and controls. PRISMA Checklist for the studies was shown as Checklist S1.

### Statistical analysis

The departure of frequencies of *hsa-miR-146a* polymorphism from expectation under Hardy-Weinberg equilibrium (HWE) was assessed by the chi-square test in controls and a *P*<0.05 was considered as significant disequilibrium. The strength of the association between the *hsa-miR-146a* polymorphism and BC risk was measured by odds ratios (ORs) with 95% confidence intervals (CIs). The significance of the pooled OR was determined by the Z-test, and *P*<0.05 was considered statistically significant. For *miR-146a* G/C, the meta-analysis examined the association between C allele and BC risk compared with that for G allele in the co-dominant model (GC versus GG, CC versus GG), dominant model (GC/CC versus GG) and recessive model (CC versus GC/GG). The heterogeneity among the studies was checked by using the chi-square based Q statistic and considered statistically significant at *P*<0.10 [22]. When *P*>0.10, the pooled OR of each study was calculated by using the fixed-effects model (the Mantel-Haenszel method, which weights the studies by the inverse of the variance of estimates) [23]; otherwise, the random-effects model (the DerSimonian and Laird method) [24] was used. To adjust for multiple comparisons, we applied step-down Bonferroni method [25], which control for familywise error rate (FWE). The significance threshold was selected to be 0.1 after the Bonferroni correction, as used in the previous studies [26], [27]. The Begg's rank correlation method and the Egger's weighted regression method were used to statistically assess publication bias (*P*<0.05 was considered representative of statistically significant publication bias) [28]. All analyses were done using STATA software, version 11.0 (STATA Corp., College Station, TX, USA), and all tests were two-sided.

## Results

### Characteristics of the studies

**Figure 1:**
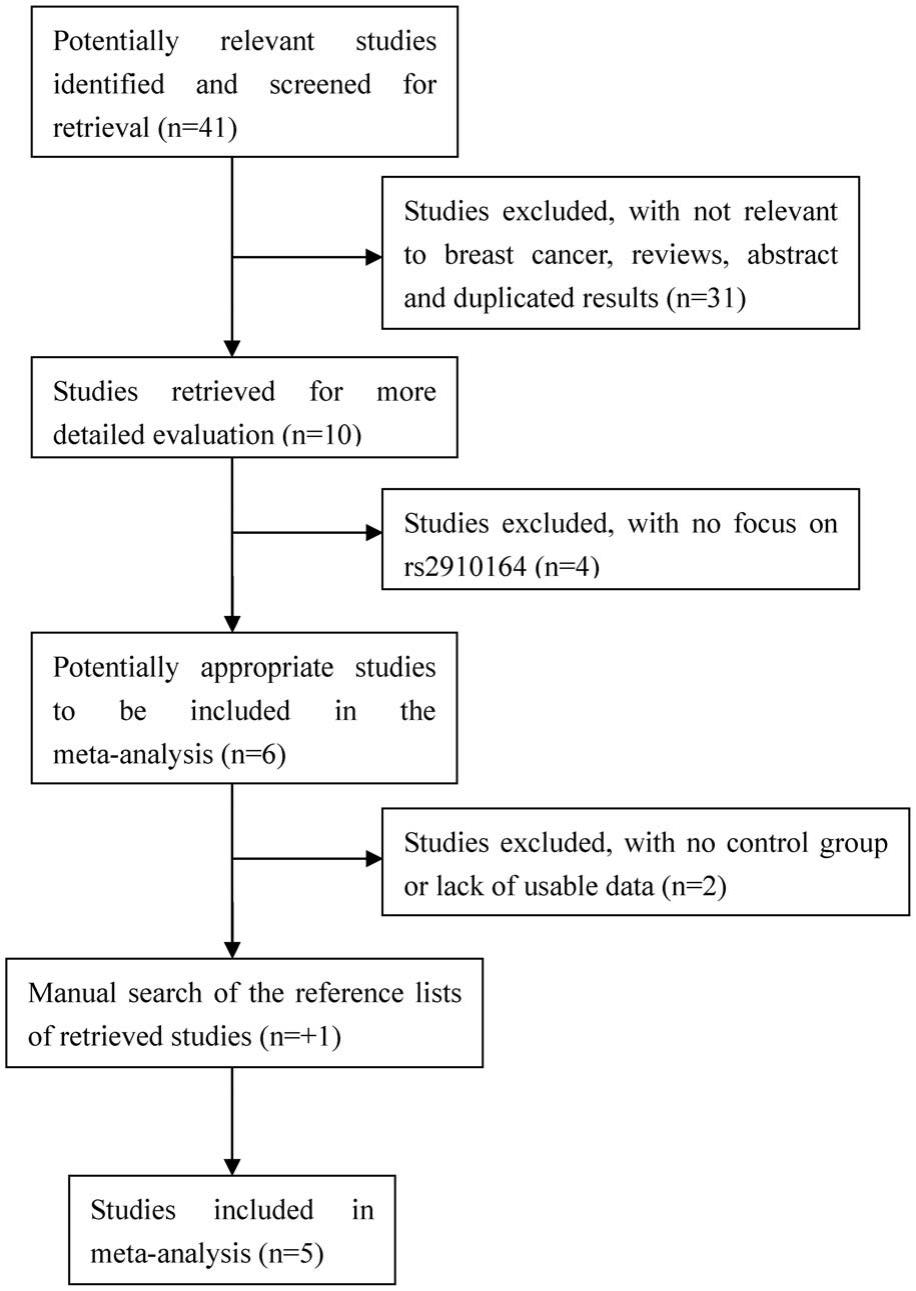
Flow chart of selection of studies and specific reasons for exclusion from the meta-analysis.

A total of 41 articles were achieved by literature search from the PubMed and EMBASE, using different combinations of key terms. As shown in Figure 1, 10 eligible studies were retrieved for detailed evaluation. We excluded six studies (four not focused on rs2910164, one was case only study and one did not present usable data). One study identified by manually searching reference lists of retrieved studies was also included [17]. Finally, a total of 6 case-control studies in 5 articles met our inclusion criteria [17], [18], [19], [20], [21], including 4238 cases and 4469 controls. Table 1 lists the characteristics of each study. There were four studies of Europeans, one study of Asians and one study of mixed populations. Genotyping methods included PCR-RFLP, TaqMan, MassARRAY and PCR-direct sequencing. The genotype distributions in the controls of all studies were in agreement with Hardy-Weinberg equilibrium except for one study [20].

**Table 1:** Characteristics of the studies included in this meta-analysis.

First author	Year	Country	Ethnicity	Genotyping methods	Sample size (case/control)	Case (%)			Control (%)			*P*_HWE_
						GG	GC	CC	GG	GC	CC	
Hu	2008	China	Asian	PCR-RFLP	1009/1093	165(16.4)	515(51.0)	329(32.6)	180(16.5)	551(50.4)	362(33.1)	0.221
Hoffman	2009	USA	Mix	MassARRAY	439/478	234(53.3)	176(40.1)	29(6.6)	273(57.1)	178(37.2)	27(5.6)	0.775
Pastrello	2010	Italy	European	Sequencing	88/155	53(60.2)	30(34.1)	5(5.7)	90(58.1)	59(38.1)	6(3.9)	0.332
Catucci	2010	Italy	European	PCR-RFLP	754/1243	409(54.2)	286(37.9)	59(7.8)	650(52.3)	520(41.8)	73(5.9)	0.019
Catucci	2010	Germany	European	PCR-RFLP	805/904	451(56.0)	304(37.8)	50(6.2)	536(59.3)	318(35.2)	50(5.5)	0.753
Garcia	2011	France	European	Taqman	1130/596	676(59.8)	388(34.3)	66(5.8)	352(59.1)	220(36.9)	24(4.0)	0.150

PCR-RFLP: Polymerase Chain Reaction-restriction Fragment Length Polymorphism; HWE: Hardy-Weinberg Equilibrium.

### Quantitative synthesis

**Figure 2:**
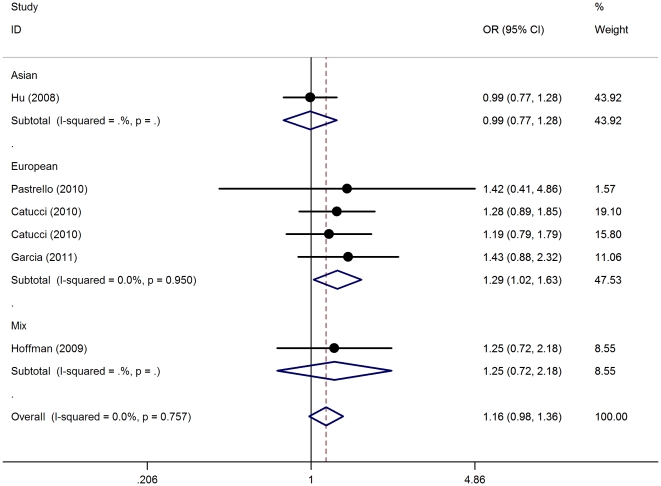
Meta-analysis with a fixed-effects model for the association between breast cancer risk and the *has-miR-146a* rs2910164 polymorphism(CC vs GG) is illustrated in subgroup analysis by ethnicity. OR: odds ratio; CI: confidence interval; I^2^, measure to quantify the degree of heterogeneity in meta-analyses.

**Figure 3:**
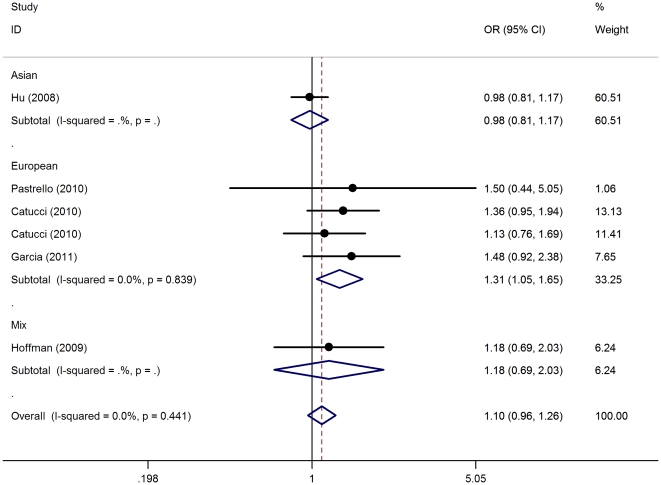
Meta-analysis with a fixed-effects model for the association between breast cancer risk and the *has-miR-146a* rs2910164 polymorphism (CC vs GC+GG) is illustrated in subgroup analysis by ethnicity. OR: odds ratio; CI: confidence interval; I^2^, measure to quantify the degree of heterogeneity in meta-analyses.

The results of the overall meta-analysis did not suggest any associations between rs2910164 polymorphism and BC susceptibility for all genetic models (for GC *vs* GG: OR = 1.00, 95% CI = 0.90−1.09, *P*_heterpgeneity_ = 0.364; for CC *vs* GG: OR = 1.16, 95% CI = 0.98−1.36, *P*_heterpgeneity_ = 0.757; for GC+CC *vs* GG: OR = 1.02, 95% CI = 0.93−1.12, *P*_heterpgeneity_ = 0.562; for CC *vs* GC+GG: OR = 1.10, 95% CI = 0.96−1.26, *P*_heterpgeneity_ = 0.441)(Table 2). When stratified according to ethnicity, we found that rs2910164 polymorphism was associated with increased BC risk among Europeans in homozygote comparison (CC vs. GG: OR = 1.29, 95%CI = 1.02−1.63, *P*_heterpgeneity_ = 0.950, *P* = 0.032) (Figure. 2) and recessive model (CC vs. GC+GG: OR = 1.31, 95%CI = 1.05−1.65, *P*_heterpgeneity_ = 0.839, *P* = 0.019) (Figure. 3). However, only in recessive model (CC vs. GC+GG: original P-value 0.019, Bonferroni P-value 0.076) remained associated to rs2910164 polymorphism and BC risk among Europeans at a significance threshold of 0.1 after Bonferroni correction.

**Table 2:** Quantitative analyses of has-miR-146a rs2910164 polymorphism on breast cancer risk.

Variables	N^a^	GC versus GG		CC versus GG		GC/CC versus GG (dominant)		CC versus GC/GG (recessive)	
		OR (95% CI)	P^b^	OR (95% CI)	P^b^	OR (95% CI)	P^b^	OR (95% CI)	P^b^
Total	6	1.00 (0.90–1.09)	0.364	1.16 (0.98–1.36)	0.757	1.02 (0.93–1.12)	0.562	1.10 (0.96–1.26)	0.441
Ethnicities									
European	4	0.96 (0.86–1.08)	0.263	**1.29 (1.02–1.63)**	0.950	1.00 (0.90–1.12)	0.427	**1.31 (1.05–1.65)**	0.839
Asian	1	1.02 (0.80–1.30)	__	0.99 (0.77–1.28)	__	1.00 (0.80–1.27)	__	0.98 (0.81–1.17)	__
Mix	1	1.15 (0.88–1.51)	__	1.25 (0.72–2.18)	__	1.17 (0.90–1.51)	__	1.18 (0.69–2.03)	__

The numbers in bold indicated statistically significant values.

a Number of comparisons.

b P value of Q-test for heterogeneity test.

### Publication bias

**Figure 4:**
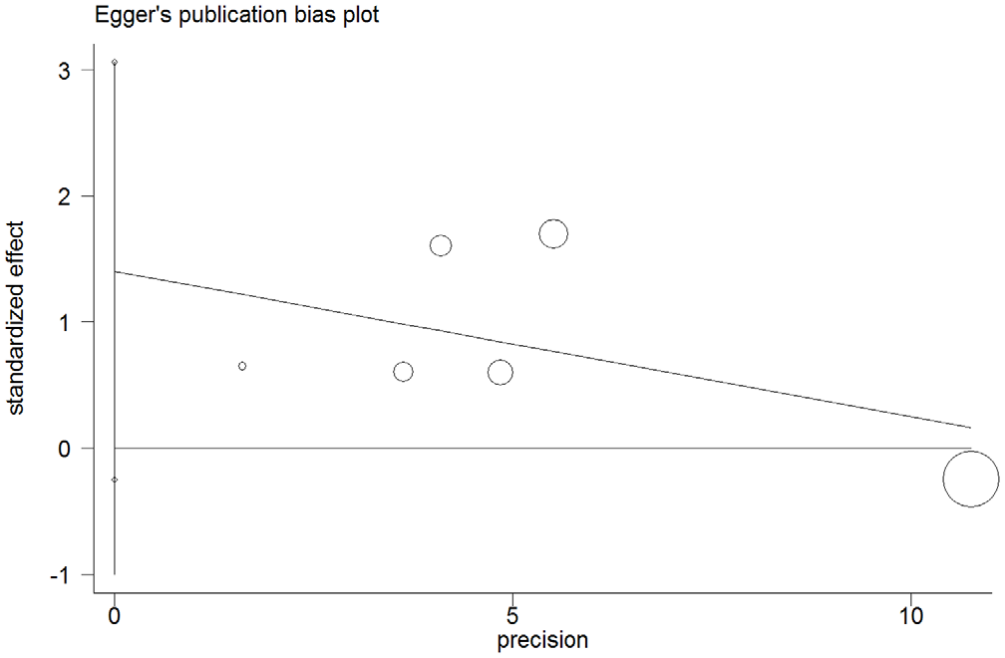
Egger’s publication bias of has-miR-146a rs2910164 polymorphism and breast cancer risk (Egger’s test for publication bias was not significant: CC vs GC+GG, P = 0.078).

Begg's rank correlation method and Egger's weighted regression method were used to assess publication bias. No evidence of publication bias was observed in any comparison model (GC vs.GG: Begg's test *P* = 0.707, Egger's test *P* = 0.955; CC vs.GG: Begg's test *P* = 0.452, Egger's test *P* = 0.130; GC+CC vs.GG: Begg's test *P* = 0.707, Egger's test *P* = 0.976; CC vs.GC+GG: Begg's test *P* = 0.707, Egger's test *P* = 0.078) (Figure. 4).

## Discussion

The identification of single-nucleotide polymorphisms (SNPs) that affect gene function or expression and contribute to BC susceptibility is important to help predict individual and population risk and understand the pathogenesis of BC. A G>C polymorphism (rs2910164) has been identified in the gene for *miR-146a*, a microRNA that binds to the 3′ UTR of BRCA1 [15], [16]. This G/C SNP (rs2910164) is located within the seed sequence of *pre-miR-146a*, which is the *miR-146a* precursor. [16]. The rs2910164 polymorphism has been associated with the risk of papillary thyroid cancer [29], hepatocellular cancer [30], esophageal squamous cell cancer [31], gastric cancer [32], [33], prostate cancer [34], [35] and glioma [36]. However, the relationship between rs2910164 polymorphism and BC susceptibility is undetermined. In 2008, shen *et al.* first reported a correlation between rs2910164 polymorphism in *miR-146a* and early cancer onset in familial BRCA1/BRCA2-negative breast/ovarian cancer patients [16]. This finding was replicated in a study based on 101 familial BC cases negative for mutations in BRCA1/2 [19], but not in a larger case-control study on 844 German and 760 Italian familial BC cases negative for mutations in BRCA genes [20]. Moreover, Hu *et al.* failed to confirm the association of rs2910164 with BC risk in Chinese women [21]. Recently, Garcia *et al.* showed that the rs2910164 SNP in the *miR-146a* gene was not associated with BC risk in BRCA1 and BRCA2 mutation carriers [18]. In this meta-analysis, we summarized current data on the association between *miR-146a* rs2910164 polymorphism and BC risk. We found that *hsa-miR-146a* rs2910164 G>C polymorphism was associated with a significantly increased risk of BC in the CC homozygote as opposed to the GG homozygote or GC/GG genotype among Europeans. These findings suggest, for the first time, that the CC homozygote of rs2910164 polymorphism may contribute to BC susceptibility.

MicroRNAs could function as tumor suppressors or oncogenes and are down-regulated or up-regulated in human tumors, including BC. Based on array data, it was previously reported that *miR-146a* was significantly upregulated in breast carcinoma tissues compared with normal breast tissues [37]. Overexpression of *miR-146a* has been reported as a signature in breast, pancreatic and prostate cancers [37], [38]. A unique example of a functional miRNA SNP is rs2910164, which is located in the 3p strand of *mir-146a*. This polymorphism involves a mispairing in the hairpin of the precursor, which leads to altered processing, lower expression of the mature sequence and predisposition to papillary thyroid carcinoma [29]. However, differences in genotype distribution have been found in a few case-control studies. Okubo *et al.* found that the rs2910164 CC genotype was associated with a significantly higher risk of gastric cancer when compared to non-cancer subjects [32]. In contrast, male individuals with GG genotype were 2-fold more susceptible to hepatocellular carcinoma (HCC) compared with those with CC genotype [30]. In addition, Guo *et al.* reported that rs2910164 genotype GG was associated with increased risk of esophageal squamous cell carcinoma compared with variant genotype CC in a Chinese Han population [31]. In this meta-analysis, significant association was found between the rs2910164 CC genotype and BC risk in Europeans. These results suggest that the molecular mechanisms underlying the genetic associations of miRNA-SNPs (mir-SNPs) with cancer are complex and the polymorphism might play a different role in different cancers.

One important property of the gene polymorphism is that their incidence can vary substantially between different racial or ethnic populations. Xu *et al.* found significant differences in the prevalence of the rs11614913 T allele and rs2910164 C allele among controls of Asian (0.544 and 0.498, respectively) and Caucasian (0.375 and 0.246, respectively) [39]. At population level, we found that individuals with rs2910164 C allele could reduce cancer susceptibility in Asians but not in Caucasians, suggesting a possible role of ethnic differences in the genetic background and the environment they lived in (unpublished data). Meta-analysis is a powerful statistical tool that provides a consensus by combining the data from diverse studies that reveal inconsistent results on the same problem. Some results of our study did not show any statistical significance. However, subgroup analysis by ethnicity showed a statistical significant result, suggesting genetic diversity among different ethnicities.

Although many previous studies have been carried out to reveal the potential associations between mir-SNPs and cancer risk, most of these studies use a candidate gene approach as most mir-SNPs have not been included in current genome-wide association studies (GWAS) designs [14]. The inclusion of mir-SNPs in future GWAS will help to unveil low-penetrance susceptible mutations.

In interpreting the current results, some limitations should be considered. First, lack of the original data of the reviewed studies limited our further evaluation of potential interactions, because the interactions between genes and between genes and environment may modulate breast cancer risk. Second, only published studies were included in this meta-analysis, unpublished data and ongoing studies were not sought, which may have biased our results. Third, all case-control studies were from Asia, Europe, and the United States. Thus our results may be applicable only to these ethnic groups. In spite of these, our meta-analysis has some advantages. First, in the present study, no obvious heterogeneity between studies was observed in overall comparisons by the Q-test. Second, according our selection criteria, the quality of studies included in our meta-analysis was satisfactory. Third, no publication bias was detected, indicating that the whole pooled results may be unbiased.

In conclusion, this meta-analysis suggests that CC genotype of *has-miR-146a* rs2910164 polymorphism is associated with an increased breast cancer risk in Europeans. Caution must be made about the interpretation of the results because of the limited sample size. Additional large case-control studies are necessary to validate our findings, especially in non-Europeans.

## Supporting Information

Checklist S1 (DOC)
